# ﻿Three new species and two newly recorded species of Tachininae from Tibet, China (Arthropoda, Insecta, Diptera, Tachinidae)

**DOI:** 10.3897/zookeys.1191.105549

**Published:** 2024-02-13

**Authors:** Ruiqing Dong, Junjian Li, Hui Yang, Chuntian Zhang

**Affiliations:** 1 Liaoning Key Laboratory of Biological Evolution and Biodiversity, College of Life Science, Shenyang Normal University, Shenyang 110034, China Shenyang Normal University Shenyang China

**Keywords:** *Leskia*, *Tachina*, tachinid, taxonomy, *Trichoformosomyia*, western China

## Abstract

During our studying of the fauna of Tibet, China, many specimens of the subfamily Tachininae (Diptera, Tachinidae) were collected and examined. Three species are described here as new to science, *Leskialatisurstyla***sp. nov.**, *Trichoformosomyiacuonaensis***sp. nov.**, and *Tachinajilongensis***sp. nov.**, and two species, *Nemoraeajavana* (Brauer & Bergenstamm, 1894) and *N.echinata* Mesnil, 1953, are newly recorded from Tibet. In addition to their descriptions, illustrations, and diagnoses, three identification keys are provided. The specimens in this study are kept in the Insect Collection of Shenyang Normal University, China (SYNU).

## ﻿Introduction

Tibet (=Xizang) Autonomous Region, China (an area of 1,228,400 km^2^, 26°50'–36°53'N, 78°25'–99°06'E) in western China is located on the main part of the Qinghai-Tibet Plateau (QTP). Zhang et al. (2014) reported that the QTP has an area of 2,542,300 km^2^ spanning 25°59'37"N to 39°49'33"N and 73°29'56"E to 104°40'20"E, from Pamir in the west to the Hengduan Mountains in the east (about 2800 km) and from the southern edge of the Himalayas in the south to the Kunlun and northern Qilian Mountains in the north (300–1500 km). The average elevation of this plateau is above 4,000 m. QTP has an alpine, low-oxygen environment and is also called the Third Pole of the Earth or the Asian Water Tower. In addition to China (Qinghai, Tibet, southwestern Gansu, western Sichuan, northwestern Yunnan, and southern and southwestern Xinjiang), the QTP is divided among northern Myanmar, Bhutan, northeastern and northwestern India, Nepal, northern Pakistan, Afghanistan, Kyrgyzstan, and Tajikistan. Along its southern and western borders are valleys which are one of the world’s 34 “biodiversity hotspots”, known as the Himalayan hotspot. Ouyang et al. (2005: 35) reported that QTP is a land of physical and ecological extremes, with the following climatic and biogeographic characteristics: alpine zone, temperate zone, subtropical zone, high Himalayas, and coniferous, valley and montane forests in in southeastern Tibet; shrub grassland in the middle and upper Yarlung Zangbu River valley in southern Tibet; alpine shrub meadow in southeastern Tibet; alpine grassland in central Tibet; mountain desert in western Tibet (Ali region); and arid alpine in northwestern Tibet.

Biogeographically, Tibet is mostly in the Palaearctic Region, but the southern valleys are at the crossroads of the Palaearctic and Oriental regions. Therefore, the tachinid fauna (Diptera, Tachinidae) of Tibet is a combination of eastern Palaearctic, Oriental, and endemic elements. O’Hara et al. (2009) catalogued 1109 valid species in 257 genera, 37 tribes, and four subfamilies of Tachinidae in China. Of these, there are 238 species in 81 genera, 13 tribes and four subfamilies, including 101 species of 22 genera, 10 tribes of Tachininae from Tibet. O’Hara et al. (2020a) listed 2112 species of Palaearctic Tachinidae, including 706 species of Tachininae. O’Hara et al. (2020b) reported 1247 valid tachinid species of 274 genera, 36 tribes, four subfamilies from China, including 269 species of 85 genera, 23 tribes, four subfamilies of Tachinidae with 97 species of 18 genera, eight tribes of Tachininae from Tibet. The phylogenetic position of the family Tachinidae has been partly resolved by Stireman et al. (2019).

During our studying the systematics and diversification of Tachininae (Diptera, Tachinidae) from the Qinghai-Tibetan Plateau, we collected many of specimens of the Tachininae (Diptera, Tachinidae) from Tibet and adjacent northwestern Yunnan in recent years. After careful examination, three species are recognized as new to science, including a species of *Leskia* Robineau-Desvoidy and a species of *Trichoformosomyia* Baranov (both in the tribe Leskiini) and a species of *Tachina* Meigen (tribe Tachinini). Two species of *Nemoraea* Robineau-Desvoidy are newly recorded from Tibet. Descriptions, illustrations, and diagnoses of the above species are given, and keys to the Chinese species of *Leskia* Robineau-Desvoidy and *Tachina* (s.s.) Meigen and of *Trichoformosomyia* Baranov are provided. The keys serve to revise the higher taxonomic classification of Tachininae.

*Leskia* Robineau-Desvoidy is a globally distributed genus with 40 described species, three of which are known to occur in China (O’Hara et al. 2020b; Li et al. 2023): *L.aurea* (Fallén) is widely distributed in the Palaearctic region, *L.flavitegula* Zhang was recently described from Chongqing and Hubei in the Oriental Region, and *L.miranda* Mesnil is distributed in the Palaearctic region of Japan and Russia and was recently found in the Oriental and Palaearctic regions of China (Li et al. 2023). *Trichoformosomyia* Baranov is a small genus with only three known species (Tachi 2013; O’Hara et al. 2020a). *Trichoformosomyia* species are distributed in the Oriental region including Oriental southern China (Guangxi, Sichuan, and Taiwan), Malaysia (Borneo), and Vietnam, and the Palaearctic region including the Russian Far East and Japan (Honshu). Novotna et al. (2009) identified and classified the Western Palaearctic *Tachina* species on the basis of male terminalia and a molecular analysis. O’Hara et al. (2020a) indicated that *Tachina* Meigen has 139 known species of the world, in four subgenera— *Eudoromyia* Bezzi (five species in the Palaearctic), *Nowickia* Wachtl (55 species in the Palaearctic, Oriental, and Nearctic), *Rhachogaster* Townsend (seven species in the Nearctic), *Tachina* Meigen (59 species in the Nearctic, Palaearctic, and Oriental regions)—and 13 species unplaced to subgenus. O’Hara et al. (2020b) reported 67 species of the genus Tachina (subgenus Nowickia with 15 species and subgenus Tachina with 51 species) in China. O’Hara et al. (2020a, b) reported that *Nemoraea* Robineau-Desvoidy has 39 known species in the Afrotropical, Australasian, Palaearctic, and Oriental regions, among which 14 species are known in China.

## ﻿Materials and methods

Specimens in the study were collected from Tibet, China. The morphological terminology and measurements used in the descriptions follow Cumming and Wood (2017) and Tschorsnig and Richter (1998). The specimens were examined with Zeiss Stemi SV11 stereomicroscopes. The digital images of heads, abdomens, and bodies of male adults were taken with a Leica 205A microscope and images were blended with Leica Application Suiter v. 4.12.0. Dissections of male terminalia were carried out following the method described by O’Hara (2002), and dissected terminalia were placed in glycerin in a small plastic tube pinned together with the source specimen. The species distribution map was generated with ArcGIS v. 10.2 (ESRI Inc.). The tachinid specimens of this study were deposited in the Insect Collection of Shenyang Normal University, Shenyang (SYNU).

## ﻿Taxonomy

### ﻿Key to Chinese species of *Leskia* Robineau-Desvoidy

**Table:** 

1	Abdominal syntergite 1+2 not medially excavate to posterior margin, without median marginal seta, tergite 5 with discal setae. Abdomen covered with some golden- yellow pruinosity. Genal height about 1/7 of eye height. Prementum 3–3.5 times as long as wide	***L.aurea* (Fallén)**
–	Abdominal syntergite 1+2 medially excavate to posterior margin, tergites each without discal seta. Abdomen at most covered with grayish-yellow or grayish-white pruinosity. Genal height about 1/4 of eye height. Prementum 3–7 times as long as wide	**2**
2	Prementum 3–5 times as long as wide. Tegula dark or yellow. Postgonite wider at middle in later view	**3**
–	Prementum at least 6 times as long as wide. Katepimeron with 4–5 hairs on anterior half. Tegula dark brown except base yellow, costal spine absent or short. Surstylus bluntly rounded apically in caudal view. Postgonite narrower at middle in later view	***L.latisurstyla* sp. nov.**
3	Prementum about 3 times as long as wide. Tegula dark except base brownish yellow, costal spine absent or weak. Surstylus bluntly rounded apically in caudal view	***L.miranda* Mesnil**
–	Prementum 4–5 times as long as wide. Tegula yellow, costal spine present, slightly shorter than crossvein r-m. Surstylus pointed apically in caudal view	***L.flavitegula* Zhang**

#### ﻿ Leskia latisurstyla

Taxon classificationAnimaliaDipteraTachinidae

Zhang & Dong sp. nov.

DDA11E07-38C0-5A00-8891-0A1A26590003

https://zoobank.org/12C15996-19EC-42AD-836B-E1BC12BFF7E7

Fig. 1

##### Material examined.

***Holotype***: China • ♂ (SYNU-E 19381); Tibet (= Xizang); Linzhi, Bayi Town; 29.7425°N, 94.3189°E; 3000 m elev.; 12.VIII.2013; Q. Wang leg. ***Paratype***: 1♂ (SYNU-E 21575; NW Yunnan; Gongshan County, Dimaluo Village; 27.7470°N, 98.6723°E; 1600 m elev.; 19.V.2007; X.Y. Liu leg.

##### Etymology.

The specific epithet is derived from the characteristically wide surstylus in caudal view of this species; it is composed from the Latin adjective *lati* and the noun *surstylus*.

##### Diagnosis.

This species is similar to *L.flavitegula* Zhang, but it is distinguished from the latter in the height of the gena, which is about 1/4 of eye height, the longer prementum, which is at least 6.5 times as long as wide apical 4/5 of postpedicel dark brown in male, and palpus which is slightly longer than antenna. Discal scutellar setae are slightly shorter than the scutellum length; anepimeral setae 2, katepisternal setae 3; tegula dark brown except for the yellow base. Surstylus wider and bluntly roundedat apex in caudal view, postgonite narrower at middle.

##### Description.

**Male.** Body length 10.0 mm.

**Figure 1.**
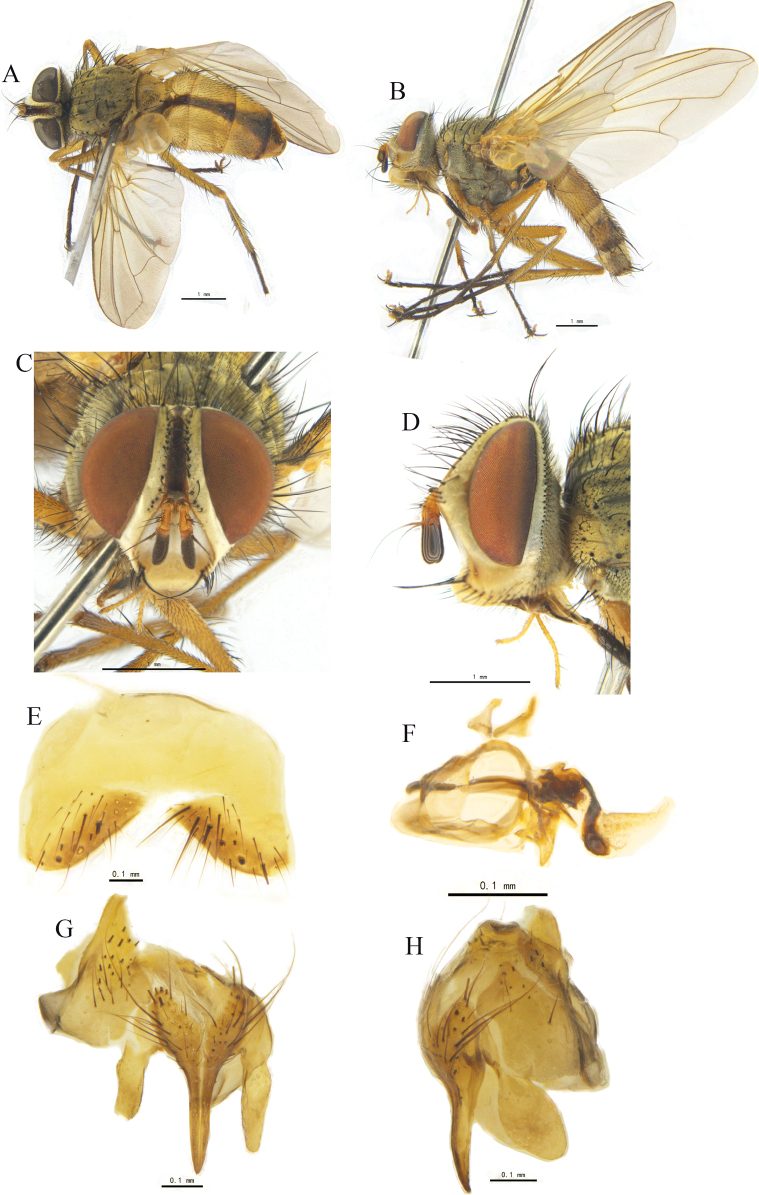
*Leskialatisurstyla* sp. nov. **A, B** ♂, bodies, dorsal and lateral views **C, D** ♂, heads, anterior and lateral views **E** sternite 5, ventral view **F** phallus (aedeagal apodeme, hypandrium, pregonite, postgonite, basiphallus and distiphallus) of male, lateral view **G, H** cerci, surstyli and epandrium of male, caudal and lateral views.

Head (Fig. 1C, D). Eye bare. Frontal vitta brownish black, ground colour of fronto-orbital plate, parafacial, and face dark yellow; fronto-orbital plate and parafacial with thin, grayish-yellow pruinosity; face with grayish-white pruinosity. Occiput with grayish-yellow pruinosity. Lunule dark yellow, with grayish-yellow pruinosity. Palpus reddish yellow on apical half and dark on basal half. Prementum gleaming black. Antenna with scape, pedicel, and basal 1/5 of postpedicel reddish yellow; apical 4/5 of postpedicel and basal 4/5 of arista dark brown. Frons slightly widened anteriorly, about 4/7 of eye width at narrowest point; frontal vitta at anterior ocellar point slightly narrower than fronto-orbital plate. In anterior view parafacial at middle about as wide as postpedicel, lower margin of face protruding forward in lateral view, vibrissa at level of lower margin of face. Fronto-orbital plate with fine hairs, parafacial bare. Genal height about 1/4 of eye height; 16–17 pairs of crossed frontal setae, with upper and lower frontal setae smaller than other frontal setae, lowest frontal setae at nearly level with apex of pedicel, 2 to many proclinate ocellar setulae, longest one slightly shorter than upper frontal setae, inner vertical setae strong, crossed, about 0.65 times as long as eye height, outer vertical seta outward, about 0.58 times as long as inner vertical seta, a pair of smaller postocellar setae. Occiput only with 2–3 rows of black hairs below postocular setae except for white hairs, and with a row of subvibrissae below vibrissa, which 0.30–0.35 times as long as vibrissa. Antenna short; scape erect, forming closed angle to pedicel; postpedicel about 3 times as long as wide and about 2.6 times as long as predicel; arista bare, thickened at least on basal 1/5. Palpus longer than antenna. Prementum 6–7 times as long as wide. Proboscis medium-sized.

Thorax (Fig. 1A, B) dark brown, with dense, grayish-yellow pruinosity on dorsum; anterior spiracle yellow; posterior spiracle reddish yellow. Thoracic dorsum with 4 dark longitudinal vittae; broad outer and narrow inner vittae on presutural scutum; distance between inner and outer vittae about 5.5 times as wide as inner vitta; inner vitta extend to scutoscutellar suture. Prosternum bare, about 1.6 times as long as wide; proepisternum bare; 3 postpronotal setae nearly arranged in a straight line; 3 presutural and 2 postsutural acrostichal setae, 3 presutural and 3 postsutural dorsocentral setae, 3 postsutural intra-alar setae, prealar seta about as long as notopleural seta, 2 supra-alar setae, anterior one stronger, 1 upward proepimeral seta, upper anepisternum with 1 shorter and upward seta, a row of 6–7 outward setae behind anepisternum, 2 anepimeral setae, a tuft of fine hairs around it, 3 katepisternal setae, 3 anterior setae set in straight line, katepimeron (= barette) with 4–5 hairs on anterior half, anatergite and katatergite bare. Scutellum with semi-erect setulae at medialy dorsal surface and 2 discal setae; apical scutellar setae crossed, upward, as long as basal scutellar setae; lateral scutellar setae slender, about 1/2 as long as subscutellar seta.

Wing pale brownish yellow, hyaline. Tegula dark brown except for yellow base; basicosta yellow. Lower calypter yellowish white, with short fringe on outer margin; halter yellowish white at middle, reddish yellow in both apexes, with 5–7 fine hairs at apical 1/4; costal spine absent or short; vein 1^st^ and 2^nd^ costal sections with fine hairs ventrally; relative lengths of 2^nd^, 3^rd^, 4^th^ costal sectors approximately as 1:2:1; base of vein R_4+5_ with 2–3 short setae dorsally and ventrally; vein M from dM-Cu crossvein to its bend about 1.3 times distance between the bend and wing posterior margin; vein M nearly parallel with vein R_4+5_.

Legs slender, yellow to reddish yellow except tarsi black-brown, with thin, grayish-white pruinosity. Fore claw and pulvillus longer than 5^th^ tarsomere, fore tibia with 2 posterior setae, 1 preapical anterodorsal seta, 1 preapical dorsal, and 1 preapical posteroventral setae. Mid tibia with 1 anterodorsal, 1 ventral, and 2 posterior setae. Hind tibia with a complete row of anterodorsal setae, 4–5 posterodorsal setae, and 3 anteroventral setae.

Abdomen long ovate, mostly yellow, with yellowish-gray pruinosity, with a dark-brown, broad, trapezoidal median marking on syntergite 1+2 and tergite 3; tergite 4 with pruinosity on basal 1/5 and dark brown on posterior 4/5; tergite 5 brown. Syntergite 1+2 medially excavate to hind margin and with a pair of lateral marginal setae; tergite 3 with a pair of lateral marginal and 2 median marginal setae; tergite 4 and 5 each with a row of marginal setae; tergites without discal setae. Posterior sternites 2–4 each with 2 posterior setae.

Sternite 5 and male terminalia as Fig. 1E–H. In ventral view, sternite 5 nearly square, V-shaped median cleft about 2/5 of sternite length; lateral lobe bluntly rounded apically. In caudal view, cerci slender and narrowed at apical 2/3 and pointed apically, and surstylus slightly longer and bluntly rounded apically. In lateral view cerci slightly bent ventrally and surstylus wide, bluntly rounded at apex. Distiphallus with some small spines on foot-like membranous part and contacted with sclerotized part. Pregonite long, postgonite short and narrow at middle.

**Female.** Unknown.

##### Distribution.

China (Tibet, Yunnan; Fig. 8).

### ﻿Key to species of *Trichoformosomyia* Baranov (revised after Tachi 2013)

**Table:** 

1	Abdomen with a longitudinal black stripe on mid-dorsal portion of syntergite 1+2–4^th^ tergite; male wing ventrally with many long setae on 1^st^ and 2^nd^ costal sectors and at base of R_4+5_	**2**
–	Abdomen without a longitudinal black stripe on mid-dorsal portion; male wing ventrally with short setae on 1^st^ and 2^nd^ costal sectors and 1–3 rather long setae at base of R4+5	***T.abbreviata* Tachi**
2	Abdominal tergites 3 and 4 without median discal setae	**3**
–	Abdominal 3 and 4 tergites each with a pair of median discal setae	***T.notata* Richter**
3	Postpronotal lobe brown to reddish yellow; basal half of postpedicel reddish yellow on inner surface; coxae and tibiae brown to yellowish; abdominal syntergite 1+2 without median marginal setae	***T.sauteri* Baranov**
–	Postpronotal lobe dark brown; antenna with postpedicel dark brown; coxae and tibiae dark brown; abdominal syntergite 1+2 with 2 median marginal setae	***T.cuonaensis* sp. nov.**

#### ﻿ Trichoformosomyia cuonaensis

Taxon classificationAnimaliaDipteraTachinidae

Zhang & Li sp. nov.

E8FF45CF-48DF-5E63-B691-93E5704BD63E

https://zoobank.org/18361DAE-8516-40B6-B09A-8C907C068F0C

Fig. 2

##### Materials examined.

***Holotype***: China • ♂ (SYNU-XZ 21006); Tibet (= Xizang); Shannan Prefecture, Cuona, Mamamenba Village; 27°52'N, 91°47'E; 2796–2850 m elev.; 3.VIII.2021; C.T. Zhang & X.Y. Li leg. ***Paratype***: 1♂ (SYNU-XZ 21007); same locality and date as holotype; JJ Li leg.

##### Etymology.

The specific epithet is derived from the locality name, Cuona County, southern Tibet, China.

##### Diagnosis.

This species is similar to *Trichoformosomyiasauteri* Baranov, which is distributed in Japan, Russia (southern Far East), China (Hunan, Guangxi, Sichuan, and Taiwan), Vietnam, Myanmar. It differs from the latter in having entirely dark-brown antennae and a dark postprontal lobe with gray pruinosity, dark-brown coxae and tibiae, abdominal syntergite 1+2 with 2 median marginal setae.

##### Description.

**Male.** Body length 9.1 mm.

**Figure 2.**
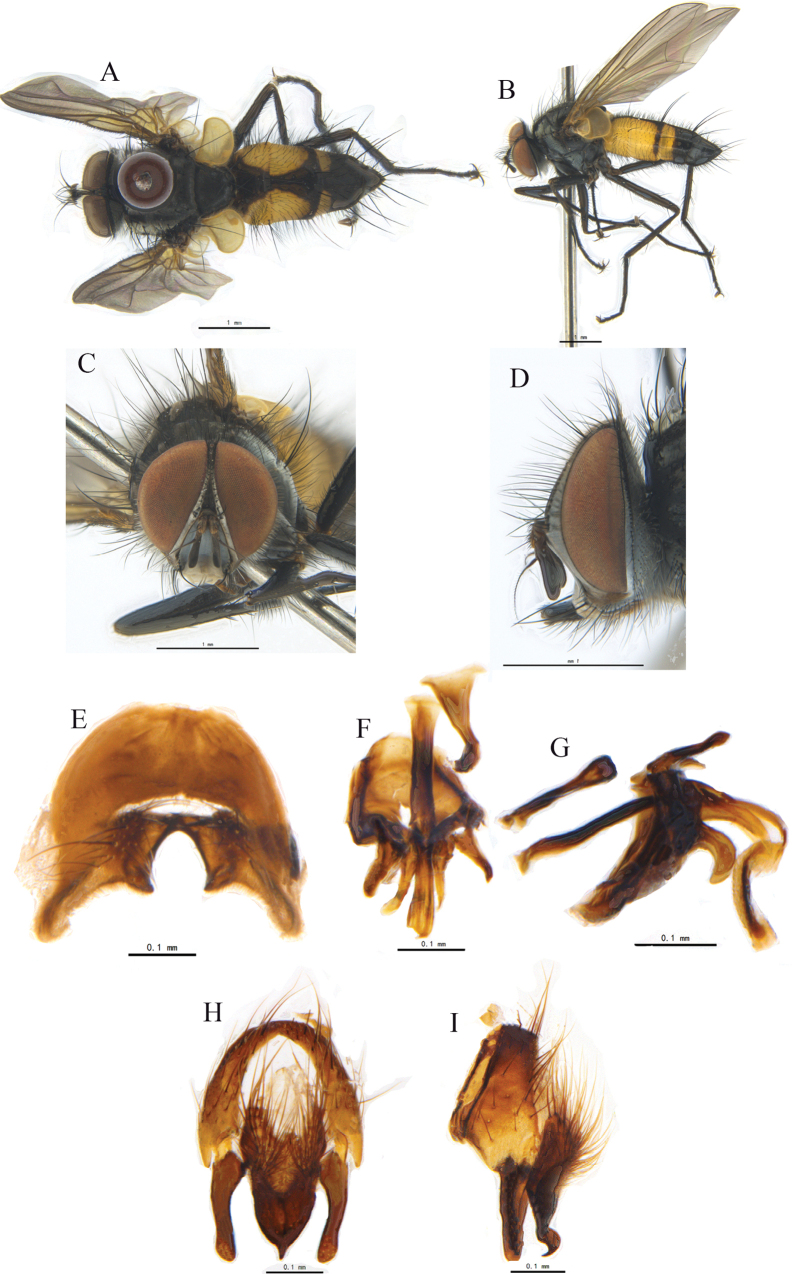
*Trichoformosomyiacuonaensis* sp. nov. **A, B** ♂, bodies, dorsal and lateral views **C, D** ♂, heads, anterior and lateral views **E** sternite 5, ventral view **F, G** phallus (ejaculatory apodeme, aedeagal apodeme, hypandrium, pregonite, postgonite, basiphallus and distiphallus) of male, dorsal and lateral views **H, I** cerci, surstyli and epandrium of male, caudal and lateral views.

Head (Fig. 2C, D). Frontal vitta linear, fronto-orbital plate, and parafacial with grayish-white pruinosity; occiput with grayish-white pruinosity. Antenna dark brown. Palpi yellow, labella yellow. Frons narrower than postpedicel width, 1/7–1/8 of eye width; genal height 1/8–1/9 of eye height or as wide as postpedicel; lower margin of face protruding forward. Vibrissa inserted at level of lower margin of face. 11–13 frontal setae, upper ones strongest, with a pair of uppermost setae strong and crossed; ocellar setae slender, hair-like. Inner vertical setae slender, hair-like; outer vertical seta hair-like, about as long as postocular setae; postocellar setae 2. Occiput mostly with white hairs except for a row of black hairs below postocular setae. Antenna with postpedicel about 3 times as long as pedicel or about 5 times as long as wide in dorsal view; pedicel with a slender seta on dorsal surface, which about twice as long as pedicel. Arista plumose; longest aristal hairs about as wide as postpedicel. Prementum 4.5–5 times as long as wide.

Thorax (Fig. 2A, B) black, with brownish-gray pruinosity; scutum with 2 broad outer and 2 narrow inner longitudinal vittae; pruinose distance between inner and outer vittae on presutural scutum about 2.5 times as wide as inner vitta; two inner longitudinal vittae on postsutural scutum combined with a broad black longitudinal vittae and extending to level of last posterior dorsocentral seta. Scutellum dark black. Postpronotal lobe black, with gray pruinosity; postalar callus dark brown. Notopleura and pleura with grayish-white pruinosity. Prosternum with hairs on sides. Postpronotal lobe with 3–4 setae arranged in a triangle; anterior 1–2 finer. One presutural and 2–3 postsutural acrostichal setae; 2 presutural and 3 postsutural dorsocentral setae, 3 postsutural intra-alar setae, 3 supra-alar setae, first one distinctly shorter than notopleural seta and first one postsutural intra-alar seta. Scutellum with three pairs of marginal scutellar setae; apical scutellar setae crossed and slightly longer than scutellum and subequal in length to basal scutellar setae; subapical scutellar setae distinctly longer than apical scutellar setae. Proepisternum bare; anepisternum with 2 upper anterior setae and a row of posterior setae; 3 katepisternal setae; anepimeral setae hair-like; katepimeron with 2–3 black hairs; katatergite bare.

Wing pale brownish, distinctly tinged with pale yellow on basal and anterior portion; tegula and basicosta dark brown. Calypters pale yellowish; lower calypter with short fringe on outer margin and not divergent from scutellum. Halter yellow. Relative lengths of 2^nd^, 3^rd^, and 4^th^ costal sectors approximately as 1:2.1:1. Base and apex of first costal sector with distinctly long setae; dorsally setulae on r_4+5_ about twice as long as setulae on first and 2^nd^ costal sectors. Base of vein R_4+5_ to vein r-m with a row of setulae ventrally and 3 setae on basal part dorsally. Bend of vein M bluntly angulated; section of M between crossveins R-M and dM-Cu longer than section between dM-Cu and bend of M, the distance between vein M from crossvein dM-Cu to bend about 2.4 times distance between bend to posterior margin of wing, or slightly shorter than distance between bend to apex of M. Crossvein dM-Cu slightly bent anteriorly. Ultimate section of wing vein CuA1 about 1/2 length of crossvein dM-Cu.

Legs coxae dark brown, with grayish pruinosity; trochanters reddish brown; femora, tibiae, and tarsi dark brown. Claws dark brown; pulvillus pale yellow. Fore claw slightly longer than 5^th^ tarsomere; fore tibia slightly longer than head height, with a row of 4–6 short, anterodorsal setae on upper 2/3; 1 posterior seta; 1 preapical dorsal seta distinctly longer than preapical anterodorsal seta; 1 preapical posteroventral seta. Mid femur with 2 anterior setae on middle and 2 preapical posterodorsal setae. Mid tibia with 1 anterodorsal, 2 posterior, and 1 ventral seta; 3 preapical dorsal setae, including 1 dorsal, 1 posterodorsal, and 1 posterior setae; 1 preapical anteroventral and 1 preapical posteroventral setae; 2 preapical posterodorsal setae. Hind femur with a row of posteroventral setae on basal half, a row of complete anterodorsal setae, 1 preapical anteroventral seta. Hind tibia with 4–5 anterodorsal setae, 2 strong, 2–3 posterodorsal setae, 4–5 ventral setae (lowest one strong), 3 preapical dorsal setae, 1 preapical anteroventral seta, without distinct preapical posteroventral seta.

Abdomen long, ovate, reddish yellow; dark brown on middle longitudinal portion of tergites, posterior 2/3 of tergite 4 and entire tergite 5; tergite 5 with grayish-white pruinosity on anterior half. Syntergite 1+2 medially excavate to 2/3–3/4, with 2 median marginal setae, with 1 lateral marginal and 3–4 lateral discal setae. Tergites each with erect hairs; tergite 3 with 2 median marginal setae, 1 lateral discal seta, without median discal seta; tergite 4 with a row of marginal setae, without median discal and lateral discal seta; tergite 5 separately with a row of marginal setae and 2 lateral discal setae. Sternite 1 with black hairs on margin.

Sternite 5 and male terminalia as Fig. 2E–I. In ventral view, sternite 5 nearly square; V-shaped median cleft about 5/9 of the sternite length; apex of lateral lobe slightly blunt and with an inner protruding and pointed apically. In caudal view, syncercus distinctly narrowed and pointed only at apex; surstylus long, thin, slightly blunt at apex. In lateral view apex of syncercus distinctly arc-like and bent ventrally; surstylus long, straight, bluntly rounded at apex; membranous and sclerotized parts of distiphallus long and narrow; pregonite long; ejaculatory apodeme large; postgonite short and arc-like.

**Female.** Unknown.

##### Distribution.

China (Tibet; Fig. 8).

### ﻿Key to Chinese species of *Tachina* (s.s.) Meigen

(revised after Mesnil 1966; Chao et al. 1998; Tschorsnig and Richter 1998; Richter 2004)

**Table:** 

1	Hind coxa with one or more setae on posterodorsal margin. Prosternum bare. Eyes bare or nearly bare. Parafacial with hairs or setulae only. Lower margin of face protruding forward. Ocellar seta proclinate, developed. Occiput without black hairs behind postocular seta row. Antenna with broad postpedicel at most as long as narrower pedicel. Arista bare. Prementum at least 4 times as long as wide. Postpronotum with >3 setae, the strongest of them arranged in a triangle. Proepisternum hary. Inner anterior surface of fore coxa bare or predominantly bare. Legs mostly yellow (at least hind tarsi) or black. Abdomen usually reddish yellow, not metallic, syntergite 1+2 medially excavated to its posterior margin	**2 (*Tachina* Meigen)**
–	Hind coxa bare on posterodorsal margin. Prosternum setose	**other genera**
2	Legs entirely black (Fig. 4A–C). Palpi inflated apically. Cerci nearly rectangular or long triangular, distinctly narrowed on apical half and blunt apex with 1–2 apical spines, and surstylus belt-shaped and bent centrally, its apex with a distinctly pointed, long tooth and without an angular lateral incision in caudal view	**Subgenus Nowickia Wachtl**
–	Legs mostly reddish yellow or black, at least hind tarsi reddish yellow. Palpi slender, cylindric. Male terminalia is a hypopygium circumversum, cerci long triangular, distinctly narrowed and pointed at apical half, with an apical spine and surstylus developed, with a distinct angular lateral incision on the apical lobe in caudal view, sternite 5 large, with a V-shaped median mid-incision at posterior margin and bluntly rounded posterior lobes (Fig. 6E–H)	**3 (Subgenus Tachina Meigen)**
3	Abdominal syntergite 1+2 without or with 2 median marginal setae (Fig. 5D–F)	**4**
–	Abdominal syntergite 1+2 with 4–12 median marginal setae (Fig. 3)	**5**
4	One presutural and 3 postsutural intra-alar setae, if 1 presutural and 2 postsutural intra-alar setae, then thorax and abdomen without pale hairs	***T.grossa* (Linnaeus), *T.punctocincta* (Villeneuve), *T.persica* (Portschinsky), *T.fera* (Linnaeus), *T.corsicana* (Villeneuve), *T.rohdendorfi* Zimin, *T.metatarsa* Chao & Zhou, *T.macropuchia* Chao, *T.magnicornis* (Zetterstedt), *T.nupta* (Rondani) (Fig. 5)**
–	One presutural and 2 postsutural intra-alar setae, pleura of thorax with pale hairs	***T.albidopilosa* (Portschinsky), *T.flavosquama* Chao, *T praeceps* Meigen**
5	Body black, with black hairs. Calypters black	***T.bombidiforma* (Chao), *T.furcipennis* (Chao & Zhou), *T.haemorrhoa* (Mesnil)**
–	Body color various. Calypters at least white or yellowish	**6**
6	Parafacial with yellow or yellowish-white hairs. Postocullar setulae usually short, not hair-like	**7**
–	Parafacial with blending black and pale hairs, or with black hairs on upper 1/2–3/4 and pale hairs on lower 1/4–1/2. Postocullar setulae slender, hair-like	**14**
7	Abdominal tergites 3 and 4 with marginal setae on ventral surface and lateral setae and laterodiscal setae	***T.pubiventris* (Chao), *T.amurensis* (Zimin), *T.tienmushan* Chao & Arnaud, *T.sobria* Walker, *T.gibbiforceps* (Chao), *T.luteola* (Coquillett)**
–	Abdominal tergites 3 and 4 without laterodiscal setae and at most tergite 3 with marginal setae on dorsal and ventral surface. Sternite 2–4 mostly with setae	**8**
8	Sternite 2–4 without setae (Fig. 3)	***T.zimini* (Chao)**
–	Sternite 2–4 with setae	**9**
9	A pair of strong apical scutellar setae straight, not crossed	***T.pingbian* Chao &Arnaud**
–	A pair of slender apical scutellar setae inclinated, crossed	**10**
10	Abdomen long ovate. Legs reddish yellow	***T.longiventris* (Chao)**
–	Abdomen ovate (Fig. 3). Femora at least black	**11**
11	Abdomen gleaming black, without pruinosity, at most with pruinosity in anterior margin on tergites 3 and 4, tergites only with brownish red hairs	***T.breviceps* (Zimin), *T.ursina* Meigen, *T.zaqu* Chao & Arnaud**
–	Abdomen wholly covered with pruinosity on dorsal surface or pruinose belts on basal 1/3–3/5 of tergiters 3 to 5	**12**
12	Tergites 3 to 5 each with distinctly pruinose belt on basal 1/3–3/5 (Fig. 3). Postpedicel about as wide as parafacial	***T.xizangensis* (Chao), *T.aurulenta* (Chao), *T.cheni* (Chao), *T.iota* Chao & Arnaud, *T.stackelbergi* (Zimin), *T.ursinoidea* (Tothill), *T.ardens* (Zimin), *T.jakovlewii* (Portschinsky)**
–	Tergites 3–5 entirely or mostly covered with pruinosity, with pruinose markings, without distinctly pruinose belt. Postpedicel narrower than parafacial	**13**
13	Three pairs of postsutural dorsocentral setae	***T.lateromaculata* (Chao), *T.subcinerea* Walker, *T.ruficauda* (Chao)**
–	Four pairs of postsutural dorsocentral setae	***T.luteola* (Coquillett), *T.pulvera* (Chao), *T.chaoi* Mesnil**
14	Body form bombylid fly-like. Basicosta dark black	***T.bombylia* (Villeneuve)**
–	Body form not bombylid fly-like. Basicosta yellow or yellowish brown	**15**
15	Abdominal gleaming black, without pruinosity	***T.breviala* (Chao), *T.breviceps* (Zimin), *T.liaoningensis* Zhang & Hao, *T.qingzangensis* (Chao)**
–	Abdomen covered with pruinose belt or pruinosity on dorsal surface	**16**
16	Tergites 3–5 without distinctly pruinose belt, at least with even pruinosity on median surface	***T.spina* (Chao), *T.medogensis* (Chao & Zhou)**
–	Tergites 3–5 with distinctly pruinose belts or pruinose markings	**17**
17	Tergites 4 and 5 each with two grayish-white rectangular lateral pruinose markings	***T.laterolinea* (Chao)**
–	Tergites with complete pruinose belt or absent, or only tergite 4 with two yellowish white lateral pruinose markings	**18**
18	Abdomen black with indistinctly dark yellow lateral markings, tergites 4 and 5 with yellowish-white hairs on dorsal surface	***T.ursina* Meigen, *T.anguisipennis* (Chao), *T.alticola* (Malloch)**
–	Abdomen reddish brown or dark brown with reddish brown on tergites 3 and 4, with broad black median vitta (male), or abdomen completely black except for apex reddish yellow (female). Abdomen only with black hairs on syntergite 1+2 and tergite 3	**19**
19	Frons of male narrower, 0.4–0.6 of eye width, parafacial wider postpedicel. Apical scutellar setae crossed, Abdominal syntergite 1+2 at most with 8 median marginal setae, tergites 4 and 5 with brownish-red hairs	***T.rohdendorfiana* Chao & Arnaud**
–	Frons of male wider, 0.9–1.0 of eye width, or 0.3–0.33 of head width, parafacial of male slightly narrower than postpedicel, scape and pedicel dark brown. Apical scutellar setae parallel, not crossed. Abdominal syntergite 1+2 with 12 median marginal setae, tergite 4 with two yellowish-white pruinose lateral markings and covered with dense, straight, yellowish-white hairs and black hairs on dorsally median portion, only tergite 5 with erect, dense, long, brownish-red hairs (Fig. 6)	***T.jilongensis* sp. nov.**

**Figure 3.**
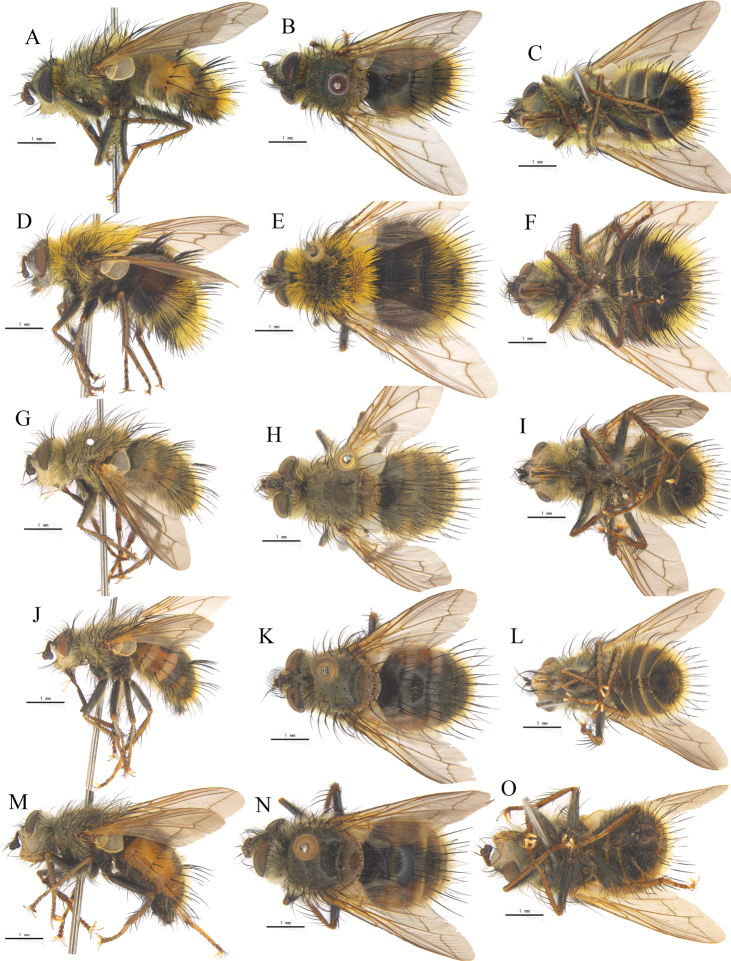
**A–C**Tachina (Tachina) anguisipennis (Chao) from Dingjie, Tibet **D–F**T. (s.s.) bombylia (Villeneuve) from Sichuan **G–I**T. (s.s.) cheni (Chao) from Yaan, Sichuan **J–L**T. (s.s.) iota (Chao & Arnaud) from Lijiang, Yunnan **M–O**T. (s.s.) zimini (Chao) from Pingwu, Sichuan, China **A, D, G, J, M** ♂, bodies in lateral views **B, E, H, K, N**, bodies in dorsal views **C, F, I, L, O** bodies in ventral views.

**Figure 4.**
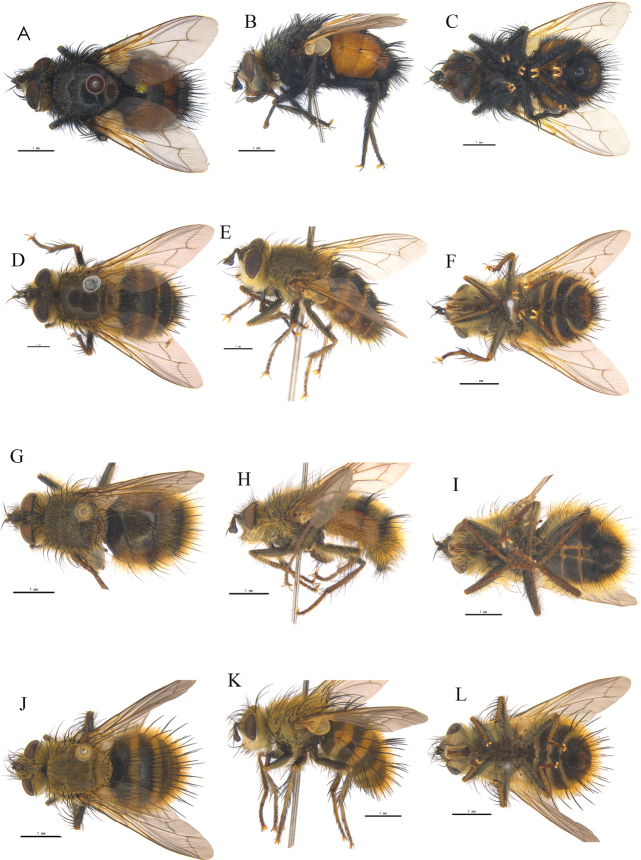
**A–C**Tachina (Nowickia) atripalpis (Robineau-Desvoidy) from Sangzhuzi, Rikaze, Tibet **D–F**T. (s.s.) amurensis (Zimin) from Dingjie, Tibet **G–I**T. (s.s.) spina (Chao) from Xiaojin, Sichuan **J–L**T. (s.s.) ursinoidea (Tothill) from Lijiang, Yunnan **A, D, G, J** ♂, bodies in dorsal views **B, E, H, K**, bodies in lateral views **C, F, I, L**, bodies in ventral views.

**Figure 5.**
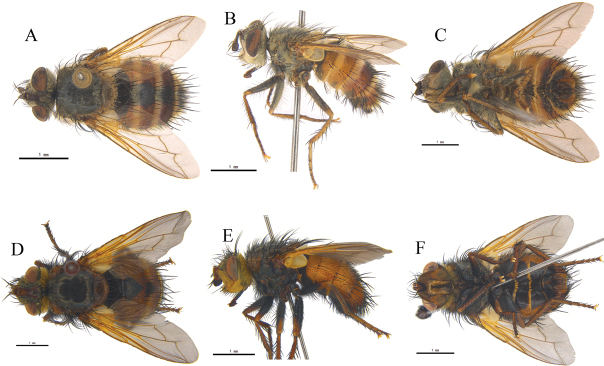
**A–C**Tachina (Tachina) sobria Walker from Yaan, Sichuan **D–F**T. (s.s.) nupta (Rondani) from Weiyuan, Gansu ♂ **A, D**, bodies in dorsal views **B, E**, bodies in lateral views **C, F**, bodies in ventral views.

#### ﻿ Tachina jilongensis

Taxon classificationAnimaliaDipteraTachinidae

Zhang & Dong sp. nov.

8C446F48-9F3E-5623-800D-E782C51D048F

https://zoobank.org/1F260668-7797-4BE2-804F-9412BFE40092

Fig. 6

##### Materials examined.

***Holotype***: China • ♂ (SYNU-XZ 210001); Tibet (=Xizang); Rikaze, Jilong County, Jipu Village, Grand Canyon; 28°22'N, 85°19'E; 2742 m elev.; 21–22.VII.2021; C.T. Zhang & X.Y. Li leg. ***Paratypes***: 3♂ (SYNU-XZ 210002 to 210004); same data as holotype • 1♂ (SYNU-XZ 210005); Tibet; Rikaze, Jilong County, Langjiu Village, Inspection Station; 28°21'N, 85°20'E; 2578–2902 m elev.; 23.VII.2021; C.T. Zhang & X.Y. Li leg.

##### Etymology.

The specific epithet is taken from Jilong County where the type locality of this species is located.

##### Diagnosis.

This species is closely similar to *T.rohdendorfiana* Chao & Arnaud, but it is distinguished from the latter in having wider frons and parafacial, narrower postpedicel in male, genal height 0.8–0.9 of eye height, apical scutellar setae parallel, abdomen with black median portion which wider than 1/3 of the tergites 3 and 4, tergites 3 with a pair of large, brownish-yellow lateral markings, tergite 4 covered with two yellowish-white lateral pruinose markings, with dense, straight, yellowish-white hairs on lateral surface and black hairs on median dorsal portion, syntergite 1+2 with 12 median marginal and 3–5 lateral marginal and laterodiscal setae, without ventral marginal seta, with black and some yellowish-white hairs on ventral surface, tergites 3 with a complete row of black marginal setae on dossal surfaces, with 1–3 laterodiscal setae and without ventral marginal seta, with black and some yellowish-white hairs on ventral surface, tergite 4 covered with two yellowish-white pruinose lateral markings, with a complete row of marginal setae and black hairs on dorsal and ventral surfaces and 3–5 laterodiscal setae, only tergite 5 entirely black, with erect, dense, long, brownish-red hairs on dorsal and ventral surface and a row of strong, black, discal setae and a row of black marginal setae on dorsal and ventral surfaces. Sternite 2 with 5–6 setae.

##### Description.

**Male**. Body length 12–14 mm.

**Figure 6.**
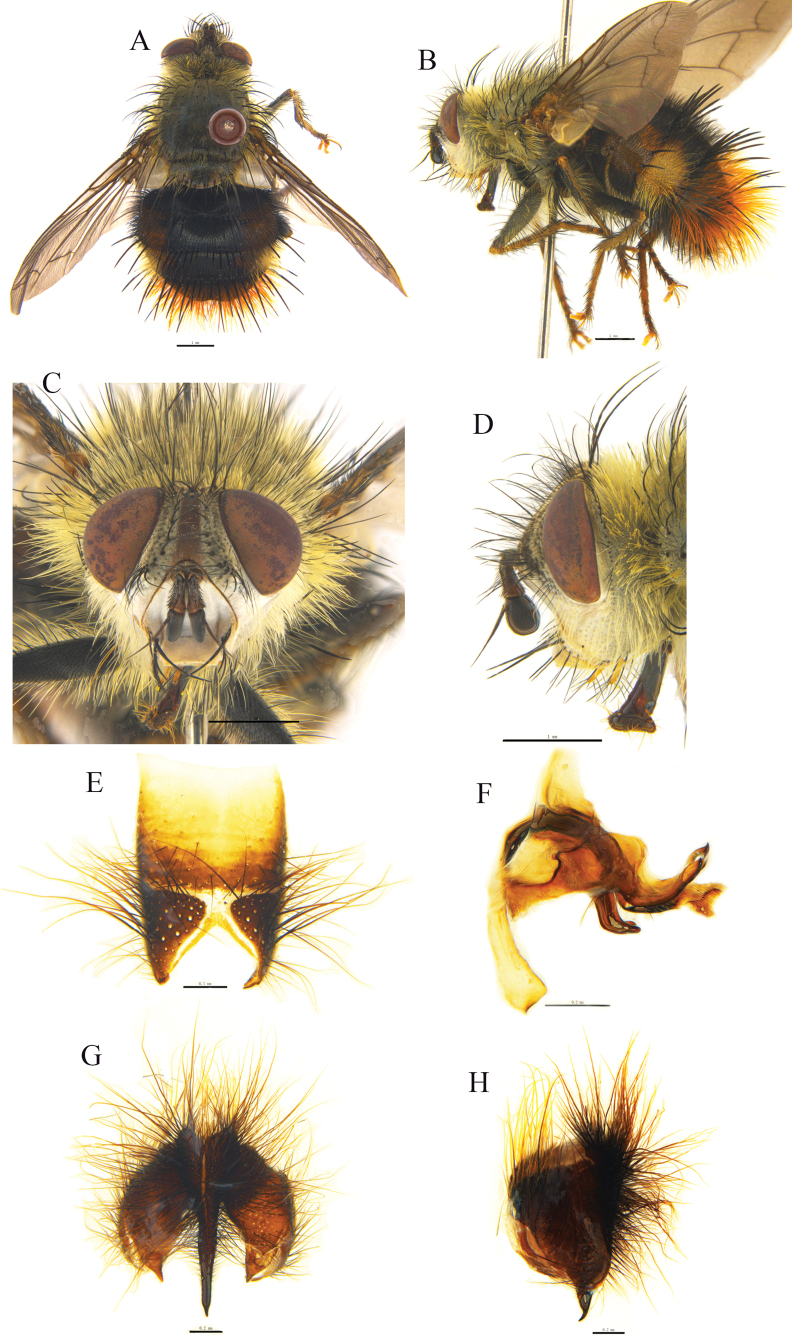
*Tachinajilongensis* sp. nov. **A, B** ♂, bodies, dorsal and lateral views **C, D** ♂, heads, anterior and lateral views **E** sternite 5, ventral view **F** phallus (aedeagal apodeme, pregonite, postgonite, basiphallus and distiphallus) of male, lateral view **G, H** cerci, surstyli and epandrium of male, caudal and lateral views.

Head (Fig. 6C, D). With grayish-white pruinosity; frontal vitta reddish brown to brown, fronto-orbital plate with pale-yellow pruinosity; lunule brown; parafacial and gena with grayish-white pruinosity; occiput with grayish-white pruinosity. Antenna with postpedicel black, with thin, gray pruinosity; pedicel brown to dark brown, with grayish-white pruinosity; base of arista dark brown to brown; palpi yellow; prementum gleaming black. Eye bare. Frons 0.3–0.33 times width of head or slightly narrower than width of eye; frontal vitta widened anteriorly, narrower than fronto-orbital plate width at narrowest point. Parafacial about as wide as postpedicel in anterior view. Genal height 0.8–0.9 of eye height; lower margin of face protruding forward; 5–6 pairs of frontal setae, with lowest setae at level with base of pedicel. Inner vertical setae strong and parallel, slightly longer than eye height; outer vertical seta outward, about 0.67 times as long as inner vertical seta; ocellar seta strong, proclinate, and about as long as upper frontal setae; a pair of smaller postocellar setae upward. Fronto-orbital plate with fine black hairs; parafacial mostly with black hairs and only white hairy on upper portion. Facial ridge with 3 setae inserted above of vibrissa. Vibrissa strong, inserted above level of lower margin of face, equal or longer than postpedicel, with a row of slender subvibrissae below vibrissae. Gena with white hairs. Occiput with long, pale-yellow hairs behind postocular setae. Postpedicel ovate, slightly narrower than parafacial and shorter than pedicel; arista bare, about as long as the combination of pedicel and postpedicel; 2^nd^ aristomere 2–2.5 times as long as its diameter. Palpi slender, longer than antenna or prementum. Prementum 4–5 times as long as wide.

Thorax (Fig. 6A, B) darkly colored, with thin, gray pruinosity; dorsum with 4 dark, longitudinal vittae; outer vittae on anterior 3/5 of postsutural scutum, inter vittae on anterior 2/5 of postsutural scutum; scutellum dark on base, reddish yellow on apical 2/3, with dense, long, yellow hairs. Anterior spiracle brownish yellow, with yellow hairs; posterior spiracle dark yellow. Thoracic dorsum densely covered with yellow fine hairs and mixed with some fine black hairs. Postpronotal lobe with 5–6 setae; 3 strong basal setae set in a triangle, 3–4 presutural and 2 postsutural acrostichal setae, 4 presutural and 4 postsutural dorsocentral setae, 1 presutural and 2 postsutural intra-alar setae, 3 strong supra-alar setae, proepisternum hairy, prosternum bare, 2 notopleural setae, meron with a row of setae, katepimeron (= barette) bare, 3 katepisternal setae. Scutellum reddish yellow except for dark base, with erect, dense, yellowish hairs, 4 pairs of marginal setae, and three pairs of discal setae along margin; apical scutellar setae standing in a straight line, not crossed, about 2 times as long as wide, and as long as subapical scutellar setae.

Wing hyaline, tinged with brownish; tegula dark brown and basicosta brownish yellow. Upper calypter pale brown except whitish anterior half; lower calypter yellowish white. Halter dark brown on apical 1/2, and brown on basal half. Costal spine absent; second costal sector of wing bare ventrally; relative lengths of 2^nd^, 3^rd^ and 4^th^ costal sectors approximately as 2.5:3.5:1; bend of vein M about right-angled; last section of vein Cu about 2/3 as long as crossvein dM-Cu; base of vein R_4+5_ with 5–6 short, black hairs on dorsal and ventral surfaces; cell R_4+5_ open.

Legs dark brown except for apex of femora reddish brown; tibiae and tarsi reddish yellow; claws reddish yellow except for dark apex; pulvillus pale yellow. Fore claws and pulvillus longer than 4^th^ and 5^th^ tarsomere combined; first tarsomere with dense, short, yellow, brush-like hairs on ventral surface. Fore tibia with a row of anterodorsal and posterodorsal setae; 2 posterior setae; mid femur with 3 anterior setae; mid tibia with a row of strong anterodorsal setae, 5 posteroventral setae, 1 strong ventral seta. Hind femur with a row of anteroventral setae, 3 preapical anterodorsal setae and 3–4 preapical dorsal setae; hind tibia with a row of irregular anterodorsal setae, 4 posterodorsal and 2 ventral setae, and apex with 2 dorsal setae, 1 anterior, 1 anteroventral, and 1 posteroventral seta.

Abdomen ovate, black on base; median depression of syntergite 1+2 extending to posterior margin; tergites 3 and 4 each with a pair of large, brownish-yellow lateral markings, and covered with dense, yellowish-white pruinose markings on tergite 4; tergite 5 gleaming black, with a shallow median depression at posterior portion. Syntergite 1+2 and tergite 3 with dense, straight, black hairs; tergite 4 with two yellowish-white pruinose lateral markings and covered with dense, straight, yellowish-white hairs, black hairs on dorsal-median portion; tergite 5 with dense, fine, brownish-red hairs. Syntergite 1+2 with 12 (6 pairs of) black, median, marginal setae, 3–5 lateral marginal setae, and 2–4 laterodiscal setae; without ventral marginal seta; with black and some yellowish-white hairs on ventral surface; tergite 3 with a complete row of 24–26 black marginal setae on dorsal surface and 1–3 laterodiscal setae, without ventral marginal seta, with black and some yellowish-white hairs on ventral surface; tergite 4 with a complete row of 30–34 marginal setae, black hairs on dorsal and ventral surfaces, and 3–5 laterodiscal setae; tergite 5 with a row of strong, black discal setae and a row of black marginal setae on dorsal and ventral surfaces. Sternite 1 with yellowish hairs; sternite 2 with 5–6 setae; sternite 3 with 6–8 setae; sternite 4 with 8 setae. Sternite 5 and male terminalia as Fig. 6E–H. In ventral view, sternite 5 nearly rectangular, with V-shaped median cleft about 1/3 of the sternite length; lateral lobe slightly pointed at apex. In caudal view, cerci slender and narrowed and pointed apically; surstylus slightly shorter and pointed apically, with a medially deep crevice. In lateral view, cerci slightly bent ventrally and pointed at apex; surstylus broad, bluntly rounded. Distiphallus with some setulae on membranous and sclerotized parts. Pregonite long and pointed apically; postgonite short and blunt apically, bent anteriorly.

**Female.** Unknown.

##### Distribution.

China (Tibet; Fig. 8).

### ﻿Newly recorded species for Xizang, China

#### ﻿ Nemoraea javana

Taxon classificationAnimaliaDipteraTachinidae

(Brauer & Bergenstamm, 1894)

6850651E-151C-580D-B96A-1A5D13824D00

Fig. 7A, C, E, G

Prodegeeria javana Brauer & Bergenstamm, 1894: 617 [also 1895: 81]. Type locality: Indonesia: Jawa, Tengger Mountains.
Prodegeeria javana : Crosskey 1976: 198. Chao et al. 1998: 2030. O’Hara et al. 2009: 160. O’Hara et al. 2020a: 741; 2020b: 946.

##### Material examined.

China – Tibet (= Xizang) • 2♀ (SYNU-XZ 210021, 210022); Rikaze, Dingjie County, Chentang Town, Xuexiongma; 2426–2600 m elev.; 27°86'N, 87°42'E; 27.VII.2021; C.T. Zhang & X.Y. Li leg.

**Figure 7.**
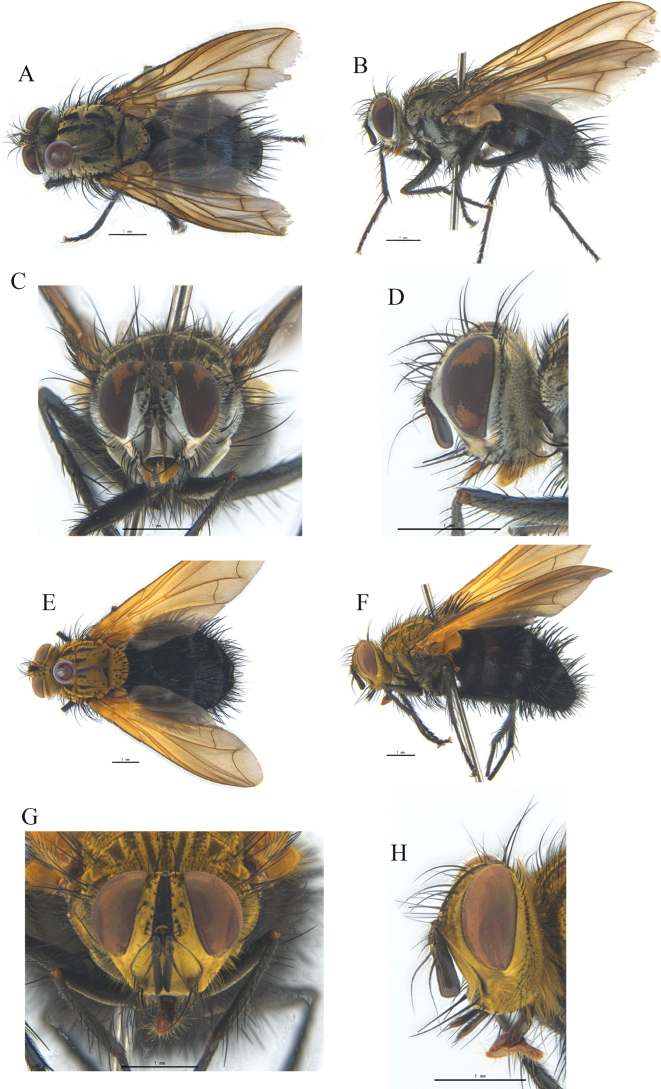
**A–D***Nemoraeajavana* (Brauer & Bergenstamm, 1895) ♀ **E–H***Nemoraeaechinata* Mesnil, 1953 ♀ **A, B**, **E, F** bodies, dorsal and lateral views **C, D**, **G, H** heads, anterior and lateral views.

##### Diagnosis.

Eye covered with dense hairs. Parafacial bare; lower margin of face protruding forward; upper part of head usually with only one row of black setulae behind postocular setae. Arista thickened at most on basal 2/5 and aristal hairs at most as long as aristal diameter; first and second aristomere each at most as long as its diameter. Palpus dark brown except yellow apex in female. Thorax with brownish-gray pruinosity. Two katepisternal setae; katepisternum and ventral surface of basal abdomen with white hairs. Wing clouded with brown along veins. Lower calypter with long hairs at least dorsally along outer margin. Inner anterior surface of fore coxa covered with appressed setulae; preapical posteroventral seta on hind tibia distinctly shorter than preapical anteroventral seta. Abdominal tergites 3–4 without median discal seta.

##### Distribution.

**Figure 8.**
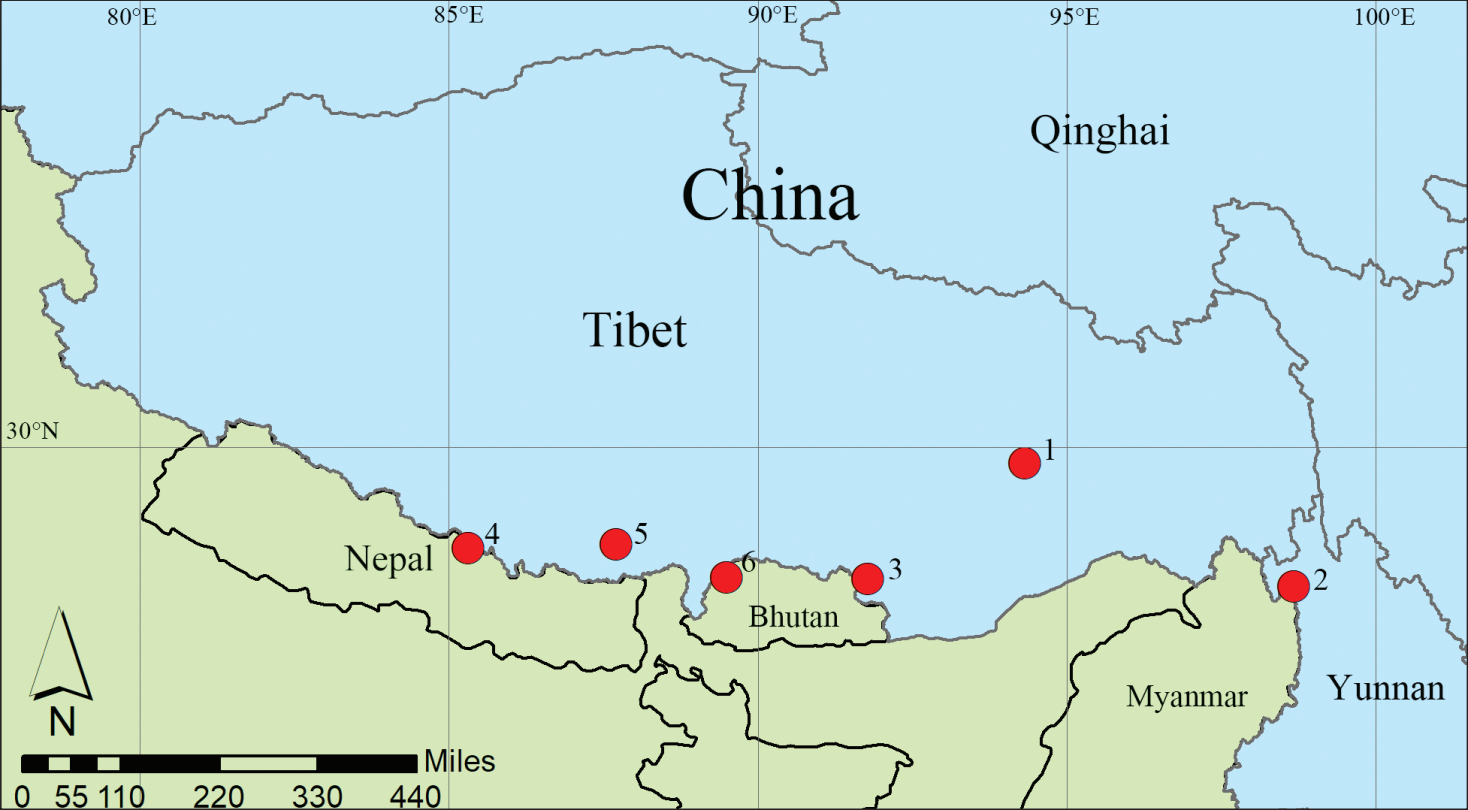
New distribution records of Tachininae from Tibet, China. 1, 2 *Leskialatisurstyla* Zhang & Dong, sp. nov. 3 *Trichoformosomyiacuonaensis* Zhang & Li, sp. nov. 4 *Tachinajilongensis* Zhang & Dong, sp. nov. 5 *Nemoraeajavana* (Brauer & Bergenstamm, 1895) 6 *Nemoraeaechinata* Mesnil, 1953.

China (Zhejiang, Hunan, Sichuan, Guizhou, Tibet; Fig. 8), Indonesia.

#### ﻿ Nemoraea echinata

Taxon classificationAnimaliaDipteraTachinidae

Mesnil, 1953

BECAB992-EEDE-5F8C-8256-A8FDFDEA4814

Fig. 7B, D, F, H

Nemoraea echinata Mesnil, 1953: 154. Type locality: Myanmar: Kachin, Kambaiti.
Nemoraea echinata : Crosskey 1976: 198. Chao et al. 1998: 2028. O’Hara et al. 2009: 159. O’Hara et al. 2020a: 741; 2020b: 946.

##### Material examined.

China – Tibet (= Xizang) • 1♀ (SYNU-XZ 210023); Rikaze, Yadong County, Yadong, Gajvsi temple; 3286 m elev.; 27°48'N, 88°90'E; 29.VII.2021; C.T. Zhang & X.Y. Li leg.

##### Diagnosis.

Head and thoracic dorsum with golden-yellow pruinosity. Eye covered with dense hairs. Frons of about 0.5 (male) or 0.6 (female) times of eye width; parafacial nearly bare; upper part of head usually with only 1 row of black setulae behind postocular setae. Antenna with postpedicel 4–5 times as long as pedicel; longest aristal hairs at most as long as aristal diameter. Presutural setae 3 and dorsocentral setae 4; apical scutellar seta absent. Lower calypter with long hairs dorsally on posterior half. Legs black. Fore tibia with 2 posterior setae; mid tibia with 5 anterodorsal and 1 ventral setae; hind tibia with 4–5 anterodorsal setae. Abdomen ovate, dark, black, with many erect setae and hairs, densely covered with gray or indistinct pruinosity on tergites. Abdominal syntergite 1+2 medially extending back to hind margin, without median marginal setae.

##### Distribution.

China (Shaanxi, Sichuan, Tibet; Fig. 8), India, Myanmar.

## Supplementary Material

XML Treatment for Leskia latisurstyla

XML Treatment for Trichoformosomyia cuonaensis

XML Treatment for Tachina jilongensis

XML Treatment for Nemoraea javana

XML Treatment for Nemoraea echinata

## ﻿Additional information

### Author ORCIDs

Ruiqing Dong https://orcid.org/0000-0001-9631-7157

Junjian Li https://orcid.org/0009-0002-1768-4800

Hui Yang https://orcid.org/0009-0007-4837-4887

Chuntian Zhang https://orcid.org/0000-0002-9514-0502

### Data availability

All of the data that support the findings of this study are available in the main text.
